# Evolutionary Tuning of Protein Expression Levels of a Positively Autoregulated Two-Component System

**DOI:** 10.1371/journal.pgen.1003927

**Published:** 2013-10-24

**Authors:** Rong Gao, Ann M. Stock

**Affiliations:** Center for Advanced Biotechnology and Medicine, Department of Biochemistry and Molecular Biology, Rutgers University - Robert Wood Johnson Medical School, Piscataway, New Jersey, United States of America; Indiana University, United States of America

## Abstract

Cellular adaptation relies on the development of proper regulatory schemes for accurate control of gene expression levels in response to environmental cues. Over- or under-expression can lead to diminished cell fitness due to increased costs or insufficient benefits. Positive autoregulation is a common regulatory scheme that controls protein expression levels and gives rise to essential features in diverse signaling systems, yet its roles in cell fitness are less understood. It remains largely unknown how much protein expression is ‘appropriate’ for optimal cell fitness under specific extracellular conditions and how the dynamic environment shapes the regulatory scheme to reach appropriate expression levels. Here, we investigate the correlation of cell fitness and output response with protein expression levels of the *E. coli* PhoB/PhoR two-component system (TCS). In response to phosphate (Pi)-depletion, the PhoB/PhoR system activates genes involved in phosphorus assimilation as well as genes encoding themselves, similarly to many other positively autoregulated TCSs. We developed a bacteria competition assay in continuous cultures and discovered that different Pi conditions have conflicting requirements of protein expression levels for optimal cell fitness. Pi-replete conditions favored cells with low levels of PhoB/PhoR while Pi-deplete conditions selected for cells with high levels of PhoB/PhoR. These two levels matched PhoB/PhoR concentrations achieved via positive autoregulation in wild-type cells under Pi-replete and -deplete conditions, respectively. The fitness optimum correlates with the wild-type expression level, above which the phosphorylation output saturates, thus further increase in expression presumably provides no additional benefits. Laboratory evolution experiments further indicate that cells with non-ideal protein levels can evolve toward the optimal levels with diverse mutational strategies. Our results suggest that the natural protein expression levels and feedback regulatory schemes of TCSs are evolved to match the phosphorylation output of the system, which is determined by intrinsic activities of TCS proteins.

## Author Summary

Different proteins are expressed at different levels that may have evolved for optimal fitness under specific environmental conditions. Additionally, cells regulate protein expression levels in response to environmental changes. For signaling proteins whose benefits are not immediately proportional to their expression levels, it is less understood whether expression levels are optimized and how protein expression levels correlate with the output responses they regulate. We developed a continuous culture competition assay to examine cell fitness at different expression levels of the *E. coli* PhoB/PhoR system. The PhoB/PhoR system, which induces expression of genes for phosphate assimilation under phosphate (Pi)-limited conditions, represents an archetype of the widely distributed prokaryotic two-component signal transduction scheme. Wild-type cells express different levels of PhoB/PhoR under different Pi conditions; these levels provide near maximal fitness under respective conditions. Under Pi-deplete conditions where PhoB-regulated gene expression is important for survival, the optimal expression level appears to correlate with the phosphorylation output of the PhoB/PhoR system. Challenging cells with expression of unfavorable levels of proteins led to diverse mutations that all shifted protein expression towards optimal levels. Our results indicate that the autoregulatory scheme and expression levels of the PhoB/PhoR system are evolved to provide optimal fitness.

## Introduction

Cells constantly face challenges from a wide variety of environmental perturbations that require evolution of appropriate mechanisms for adaptive responses. Cellular adaptation is often through modulation of gene expression that benefits cells under specific conditions. However, expressing proteins using cellular resources carries a fitness cost. Hence, evolutionary adaptation relies on development of proper signaling and gene regulatory schemes to produce appropriate amounts of proteins under particular environmental conditions, balancing cost and benefit to maximize fitness. Bacteria use the two-component system (TCS) as one of the major signal transduction schemes to respond to environmental cues. A sensor histidine kinase (HK), whose autokinase, phosphotransferase and/or phosphatase activities can be tuned by input signals, adjusts the phosphorylation level of its cognate response regulator (RR), ultimately determining output responses, mostly via transcriptional regulation [1]–[3]. Naturally, not only the physico-chemical properties but also the quantities of TCS proteins can influence the output, and thus could be subject to evolutionary optimization. Adaptation to various environments requires appropriate expression levels of TCS-regulated genes as well as genes encoding TCS proteins themselves to provide fitness advantages. How different environments shape the fitness profile and select particular TCS quantities remains largely unknown.

In many cases, the quantities of HK and RR are autoregulated. In *E. coli*, nearly half of the 30 RR transcription factors auto-activate expression of operons encoding themselves [4]. Genetic mechanisms and regulatory features of this feedback control have been explored [4]–[8] but the potential fitness benefit of TCS autoregulation in environmental adaptation are less examined experimentally. Positive feedback can lead to an ultra-sensitive switch-like response, an increase of regulatory capacity, a delay of response time, and promotion of a bistable system that can yield all-or-none output [4], [5], [9]. The ability to switch between two discrete “ON” and “OFF” states or to defer responses after multiple cell division cycles could be beneficial to some differentiation and developmental processes [10]–[12]. Elimination of the positive feedback has been shown to affect regulatory and temporal precision of these developmental processes [12], [13]. However, a binary or greatly delayed response is not likely preferred by all signaling systems; rather, a continuous well-defined output in relation to input signals allows cells to make accurate and prompt adjustments. It has been suggested that most TCSs tend to be monostable and the TCS autoregulatory architecture is distinct from conventional positive feedback loops due to the negative phosphatase activities of bifunctional HKs [6], [7], [14]. Constitutively expressed TCSs often complement the loss of autoregulated systems without causing apparent differences in steady-state outputs under laboratory conditions [7], [15]–[19]. This raises the question of what evolutionary advantages autoregulation brings over constitutive expression in these systems and demands a comprehensive mapping of cell fitness at different TCS expression levels to understand the evolution of TCS autoregulation.

**Figure 1:**
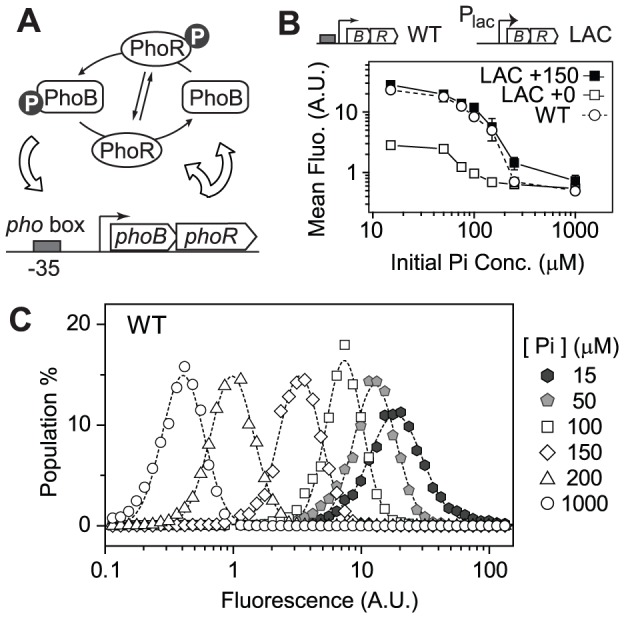
Autoregulation of PhoB/PhoR amplifies the graded output response. (A) Positive autoregulation of the PhoB/PhoR system. (B) PhoB-regulated transcription output of autoregulated (WT) and non-autoregulated (LAC) strains. RU1465 (WT) and RU1653 (LAC) carrying a *phoA-yfp* reporter were grown in MOPs medium with indicated initial Pi concentrations. Depletion of Pi led to activation of the *phoA-yfp* reporter and mean fluorescence of ∼20000 cells was determined. IPTG concentrations of 0 and 150 µM were used to induce PhoB expression in the LAC strain to achieve two different constant levels, corresponding to basal and autoregulated WT PhoB concentrations, respectively (Table S1). Error bars are SDs of three independent experiments and unseen error bars are smaller than symbols. (C) Population distribution of single-cell output responses of RU1465 (WT) at indicated initial Pi concentrations. Dashed lines represent lognormal fit of cell population distribution.

Fitness benefits of expressing TCS regulators rely upon the concerted transcription of ensemble of TCS-regulated output genes. The fitness landscape may well be correlated with TCS concentration-dependent output profiles. Intuitively, selection should produce TCSs that in environments of stimulation will have optimal concentrations of TCS proteins to elicit sufficient output to offset the cost of TCS expression. Autoregulation reflects the need for different optimal TCS levels in different environments. To examine the dependence of fitness on TCS expression levels and output responses, the autoregulated *E. coli* PhoB/PhoR system was used as a model system because the relation between its output phosphorylation profiles and protein levels has been well defined [19]. The PhoB/PhoR system activates genes responsible for assimilation of phosphorus in response to limitation of environmental phosphate (Pi) concentrations [20], [21]. Phosphorylated RR, PhoB, also binds to the *pho* box at its own promoter and stimulates the expression of PhoB and PhoR (Figure 1A). A ∼20 fold increase of PhoB concentration has been observed upon Pi-depletion. Replacing the autoregulatory *phoB* promoter with IPTG-inducible promoters allowed the characterization of phosphorylation profiles at different PhoB/PhoR levels [19].

Here we report comparison of output responses of individual cells for the autoregulated and constitutively expressed PhoB/PhoR system to examine whether bistability exists for the autoregulated WT system and how autoregulation contributes to cell fitness. The correlation of output to PhoB/PhoR levels prompted a thorough examination of cell fitness at different constitutive PhoB/PhoR levels in the absence and presence of stimuli. A competition assay in continuous cultures was developed for revealing the dependence of cell fitness on phosphorylation output and PhoB/PhoR expression levels. Under Pi-replete conditions high constitutive expression of PhoB/PhoR caused a decrease in fitness. In contrast, under Pi-deplete conditions, cell fitness peaked at an expression level close to the wild-type (WT) PhoB/PhoR concentration, at which the phosphorylation output starts to plateau. Further increase of expression apparently no longer offers sufficient advantages to overcome the cost of protein expression. To investigate whether bacteria can tune their expression levels to optimality through evolution, laboratory evolution experiments were performed for cells with unfavorable amounts of proteins under specific Pi conditions. These conditions led to evolution of diverse mutants that all shifted protein expression toward optimal levels to have greater fitness. Our results demonstrate that expression levels of TCS proteins are evolutionarily optimized and the autoregulatory scheme of the PhoB/PhoR system allows cells to adapt to both Pi- replete and -deplete conditions by expressing different optimal levels of TCS proteins to balance the cost and benefit.

## Results

### Graded Response for the Autoregulated PhoB/PhoR System

To examine output responses of single cells, the gene encoding yellow fluorescent protein (*yfp*) was fused to the promoter of the PhoB-regulated gene *phoA* and placed into the chromosome. The resulting strain still carries the original copy of *phoA* encoding an alkaline phosphatase (AP) and showed identical AP response curves to Pi concentrations as the WT strain (Figure S1A). YFP fluorescence followed a similar increasing trend as AP activities when the starting Pi concentration in the medium decreased (Figure 1B). Once the *phoBR* operon was placed behind a non-autoregulatory *lac* promoter in the LAC strain, mean fluorescence output clearly depended on expression levels of PhoB and PhoR (Figure 1B). In the absence of IPTG, PhoB concentration of the LAC strain is at a low level similar to the uninduced WT level observed under Pi-replete conditions (Table S1) and fluorescence output is correspondingly limited. The presence of 150 µM IPTG resulted in a level of PhoB comparable to the autoregulated WT level under Pi-deplete conditions and the output response curves were also comparable. It appears that positive autoregulation allows auto-amplification of PhoB and PhoR levels, leading to amplification of output responses. Analyses of single-cell fluorescence indicated that all strains, constitutive or autoregulated, displayed a graded dependence of output on Pi concentrations (Figure 1C and Figure S1). Bistability, or bimodal distribution of responses, was not observed under experimental conditions. At the timescale of the experiments, no significant delay of response time was observed for the autoregulated PhoB/PhoR system because the level of phosphorylated PhoB (PhoB∼P) was shown to increase with comparable response time in either the autoregulated WT strain or the constitutive LAC strain [19].

### Dependence of Cell Fitness on TCS Expression Levels

**Figure 2:**
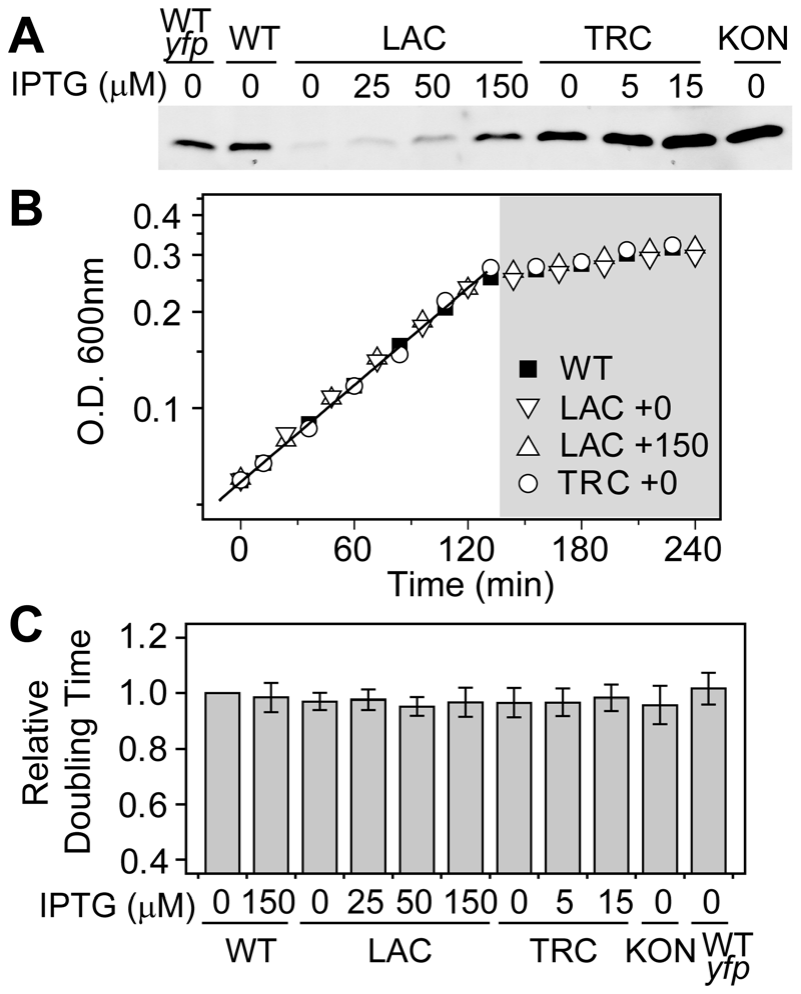
Growth rates in batch cultures are similar for the autoregulated WT and constitutive strains. (A) Western blot analyses of PhoB expression. Wild type strains BW25113 (WT) and RU1622 (WT-*yfp*), IPTG-inducible strains RU1616 (LAC) and RU1618 (TRC), and the constitutive strain RU1617 (KON) were grown to Pi-depletion under different IPTG concentrations and assayed for PhoB expression. Growth curves (B) and doubling times (C) of strains with different PhoB levels. Indicated strains were grown in MOPS media with an initial Pi concentration of 50 µM. Depletion of Pi accompanies the change of the growth rate and cells quickly enter into the stationary phase (shaded). Numbers after the “+” sign indicate IPTG concentrations. Relative doubling times were determined by fitting the exponential growth curves with an exponential function and compared to the doubling time of the WT strain. Results are shown as the mean and SD of at least four independent experiments.

The observed concentration-dependent output differences may have a direct consequence on cell fitness, which is often assessed by comparing cell growth rates under various conditions. Despite different constitutive levels of PhoB achieved in the LAC, TRC or KON strains whose *phoBR* promoter was replaced by IPTG-inducible (LAC and TRC) or constitutive (KON) promoters (Figure 2A and Table S1), cells displayed similar growth curves in MOPs medium, indistinguishable from the autoregulated WT strain (Figure 2B). As reported previously [22], [23], Pi-depletion causes a halt of the logarithmic growth and cells enter into a Pi-limited stationary phase. There is minimal difference in optical densities within the Pi-limited growth phase for these strains. Constitutive expression of the PhoB/PhoR TCS proteins also did not result in significant growth defects for the logarithmic growth phase and all shared similar doubling times (Figure 2C). Therefore, it appears that either the autoregulated WT strain does not confer fitness advantages over constitutive strains or the fitness costs of constitutively high expression are too small to be manifested under experimental conditions.

**Figure 3:**
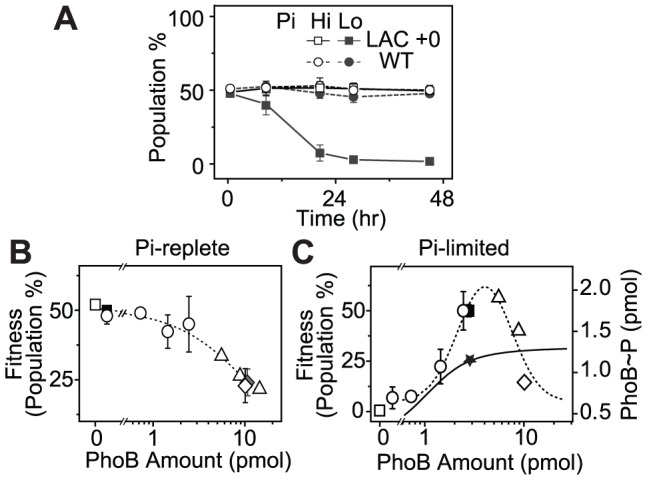
PhoB expression levels correlate with cell fitness in continuous competition assays. (A) Non-fluorescent bacteria population in continuous cultures when competed against WT-*yfp* (RU1622). A 50∶50 mixture of WT-*yfp* with indicated strains were grown in continuous cultures. Pi-replete and -deplete conditions were achieved with different Pi concentrations, 300 µM (Hi) and 12 µM (Lo), in the fresh medium supplied through the chemostat inlet. Bacteria population at 24 h was used to evaluate cell fitness under Pi-replete (B) and -deplete (C) conditions. Different PhoB levels were achieved with different IPTG concentrations using the following strains: BW25113 (WT, solid square), RU1631 (Δ*phoB*, open square), RU1616 (LAC, open circle), RU1617 (KON, open diamond), RU1618 (TRC, open triangle) and RU1619 (KON *phoB* ^D53A^, solid diamond). PhoB amount was determined previously from 0.3 OD_600_*ml of cells [19]. A value of 3 pmol in these cells is estimated to correspond to a PhoB concentration of ∼10 µM. Dotted lines represent linear and lognormal trend lines under respective conditions. The solid line represents the saturating dependence of output PhoB∼P levels on total amount of PhoB measured previously [19]. The star marks the PhoB∼P level at the autoregulated WT concentration of PhoB. Error bars are SDs and the number of independent experiments is documented in Table S1.

Batch cultures in MOPs medium only allowed approximately 2 h of logarithmic growth before reaching the stationary phase, which may not be adequate to reveal any growth differences. To examine potential fitness costs and benefits, bacteria competition was assayed in continuous cultures to allow prolonged constant growth. Because bacteria growth in continuous cultures is significantly different from that in batch cultures and bacteria consumed different amount of Pi in batch and continuous cultures to reach different O.D., AP activities were used to define the activating Pi-deplete and inactive Pi-replete conditions in continuous cultures (Figure S2). All strains were competed against a *yfp*-carrying WT strain, WT-*yfp*, which allows determination of the population distribution by cellular fluorescence. Starting from a 50∶50 mixture, the population of the non-fluorescent WT strain remained constant under both Pi-deplete and -replete conditions (Figure 3A), suggesting an equal fitness for WT and WT-*yfp* under both experimental conditions. In contrast, the constitutive LAC strain that expresses PhoB at a low level displayed a fitness dependence on Pi concentrations. Under the Pi-replete condition, LAC is equally fit as WT-*yfp*, while a gradual decrease in population was observed under the Pi-deplete condition, presumably due to its limited output response. After 24 h, the population of the LAC strain decreased to less than 1% of the total bacteria population.

Bacteria populations after 24 h of competition against WT-*yfp* were thus used to evaluate cell fitness at a range of PhoB expression levels (Figure 3B, 3C and Table S1). Strains with no or low PhoB expression, such as the *phoB* deletion or the WT strain, had high fitness under Pi-replete conditions. Increasing PhoB/PhoR concentrations reduced cell fitness (Figure 3B). Because high levels of PhoB/PhoR can promote a modest increase of expression of PhoB-activated genes in the absence of stimuli [19], fitness reduction can be attributed to cost of protein production as well as activities of proteins encoded by these PhoB-activated genes, which are tailored for Pi-depleted environments and detrimental under Pi-replete conditions [20], [24], [25]. However, the elevated basal activity of PhoB at high expression levels is dependent on the conserved D53 residue in PhoB [19] yet the D53A mutant showed similar fitness to the constitutive KON strain without the mutation, arguing against the protein activities as a main cost of fitness.

When the system is stimulated under Pi-deplete conditions, cell fitness followed a different pattern of dependence on TCS expression levels (Figure 3C). Cells with no or low PhoB expression were unable to compete with WT-*yfp* that expressed PhoB at a high level through autoregulation. For the same LAC strain, increasing IPTG concentrations raised PhoB levels as well as the output responses. Correspondingly, the fitness of bacteria increased until the PhoB concentration became comparable to the WT level. Further increase of PhoB levels eventually led to diminishing fitness of cells. As discovered previously [19], the output of the system, indicated by the concentration of phosphorylated PhoB (PhoB∼P) shown on the right axis of Figure 3C, increased along with PhoB/PhoR levels and started to saturate around the WT concentration of PhoB (solid line, Figure 3C). Thereafter, increase of PhoB concentration does not enhance the beneficial output further but still carries great costs of protein production under Pi-depleted conditions, reducing the overall cell fitness. It appears that the WT level of PhoB has been optimized to provide close to maximal fitness under Pi-deplete conditions.

### Evolutionary Tuning of PhoB/PhoR Levels under Pi-Deplete Conditions

**Figure 4:**
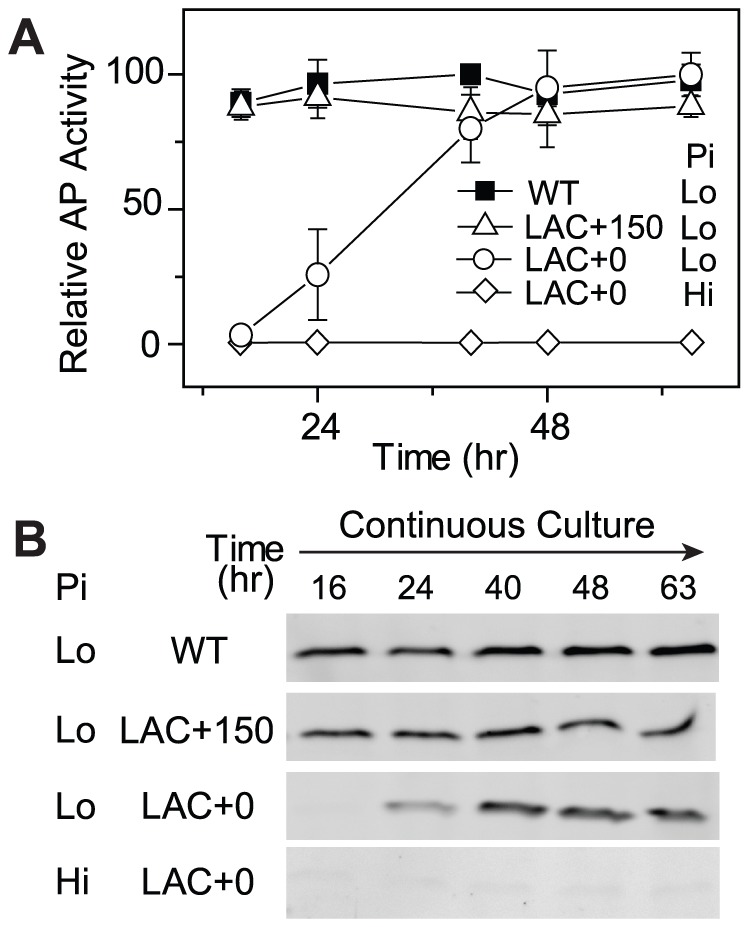
Cells expressing low levels of PhoB adapt to Pi-deplete environments by increasing PhoB expression. Time-dependent AP activities (A) and PhoB levels (B) are shown for continuous cultures with indicated strains, BW25113 (WT) and RU1616 (LAC). Pi concentrations of 300 µM (Hi) and 12 µM (Lo) in the inlet fresh medium were used for Pi-replete and -deplete environments, respectively. Numbers after the “+” sign indicate IPTG concentrations. 0.3 OD_600_*ml of cells collected from the chemostat outlet flow were used for analyses of AP activities and PhoB expression. Error bars are SDs of three independent experiments and unseen error bars are smaller than symbols.

The optimal PhoB level of WT is likely a result of adaptive evolution to balance costs and benefits. For a given *E. coli* strain with non-optimal expression of PhoB and reduced fitness, such as the LAC strain in the absence of IPTG (LAC_0_), do bacteria actually evolve toward the optimal expression level? We tested this by following laboratory evolution of bacteria in Pi-limited continuous cultures. At the start of the continuous culture, the LAC strain in the absence of IPTG displayed an extremely low level of output in AP activity as well as minimal PhoB levels (Figure 4). Only after 48 h of growth, approximately ∼18 generations, AP activity rose to a level comparable to that of the WT strain and the PhoB expression level reached the optimal level observed for the WT strain. This adaptation is clearly dependent on stimuli because PhoB concentration and AP activity remained constantly low when Pi was replete. On the other hand, when 150 µM IPTG is present in continuous cultures (LAC_150_), LAC displayed an optimal PhoB level, high output and high fitness that are all comparable to WT, thus no further increase of PhoB level or AP activity were observed.

**Figure 5:**
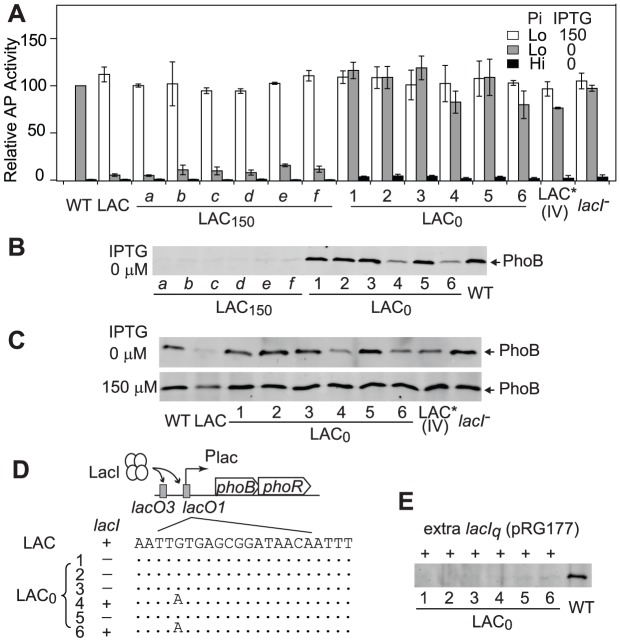
Adapted LAC_0_ cells carry promoter regulation mutations for optimal expression of PhoB. AP activities (A) and PhoB levels (B) of individual colonies isolated from LAC_0_ and LAC_150_ cultures. Indicated strains were grown in batch cultures with 50 µM (Lo) or 2 mM Pi (Hi) for 3 h. PhoB levels are shown for cells grown in Pi-replete medium (2 mM) except for WT. Error bars are SDs of at least four independent experiments. (C) IPTG-induced PhoB expression of indicated strains under Pi-deplete conditions. (D) Schematic representation of adaptive mutations. In the LAC strain, LacI binds to *lacO* regions at the *lac* promoter before *phoBR*. The presence or absence of *lacI* is indicated by “+” or “−” symbols. Sequences of the *lacO*1 site from adaptive mutants are shown with dots representing identical sequences. (E) Repression of PhoB expression by additional copies of *lacI*. Indicated colonies were transformed with the *lacI_q_*-containing plasmid pRG177 and probed for PhoB expression in the absence of IPTG.

To investigate mechanisms behind the observed adaptation in the LAC_0_ continuous culture, individual colonies were isolated to compare their PhoB expression levels and output responses to the original LAC strain in batch cultures (Figure 5). Not surprisingly, as bacteria in LAC_150_ cultures can manage adequate output responses with sufficient levels of PhoB, colonies isolated from LAC_150_ cultures showed similar outputs in AP activity to the original LAC strain, implying the absence of mutations in the PhoB/PhoR pathway. In contrast, all six colonies isolated from LAC_0_ cultures showed elevated AP activities even in the absence of IPTG while the low PhoB concentration of the original LAC strain under the same condition only gave limited output (Figure 5A). Increased AP activities are not the result of stimuli-independent constitutive activation of *phoA* expression, and all colonies maintained low AP activities under Pi-replete conditions. Among six LAC_0_ colonies, colony 4 and 6 showed slightly lower AP activities while other colonies had high AP activities, similar to WT, even in the absence of IPTG. Correspondingly, colonies 4 and 6 showed sub-optimal levels of PhoB while other LAC_0_ colonies expressed *phoB* at the optimal WT level in the absence of IPTG (Figure 5B). The *lac* promoters before *phoB* in colonies 1, 2, 3 and 5 appeared to be no longer regulated by IPTG and the regulation in colonies 4 and 6 was weakened with a high basal expression (Figure 5C). The adaptation observed in LAC_0_ cultures occurred via altering promoter regulation that shifted the PhoB concentration toward the optimal level.

The *lac* promoter is repressed by LacI through binding to *lacO* regions of the promoter. Therefore the *lac* promoter, *lacI* and *phoB* were sequenced to map the adaptive mutations. Consistent with the fact that PhoB expression and AP activity profiles of LAC_150_ colonies were similar to those of the original LAC strain, no mutations were found in these regions for colonies of the LAC_150_ culture. In contrast, colonies 4 and 6 isolated from the evolved LAC_0_ culture carry a G to A mutation in the *lacO*1 region at the promoter of *phoBR* while *lacI* appeared to be missing in other LAC_0_ colonies (Figure 5D). No mutations were identified in the coding sequences of *phoB* for any colonies. Re-introduction of the same G to A mutation into the *lac* promoter (LAC* IV) resulted in identical AP activity profiles and PhoB expression levels observed for LAC_0_ colonies 4 and 6 while deletion of *lacI* (*lacI* ^−^) in the LAC strain gave the same phenotype as other LAC_0_ colonies (Figure 5A and 5C). Further, introduction of an additional plasmid-encoded *lacI_q_* into LAC_0_ colonies re-established repression of the *lac* promoter and suppressed expression of *phoB* (Figure 5E). Combined, the above analyses indicate that LAC_0_ cells evolved to express *phoB* at or close to the optimal level by mutating different promoter regulatory elements that allowed them to generate sufficient output responses to survive under Pi-deplete conditions.

**Figure 6:**
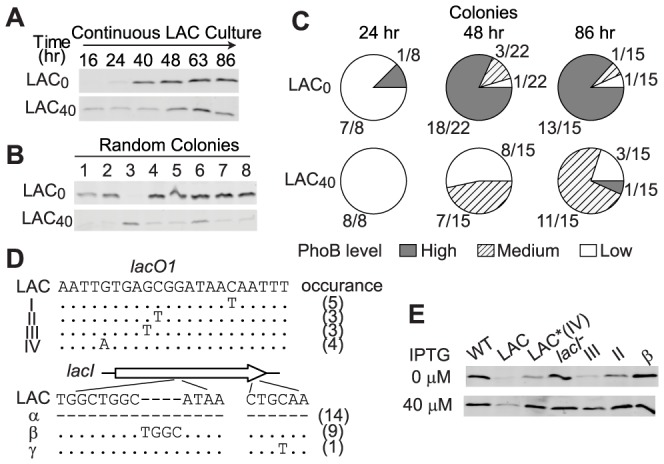
Intermediate PhoB levels lead to a slower adaptation pace and distinct adaptive genotypes. (A) PhoB levels of the indicated adapting cultures. IPTG concentrations of 0 µM and 40 µM were included in the medium to give low and intermediate initial PhoB levels, both of which were lower than the optimal concentration. (B) PhoB expression in batch cultures from random colonies isolated from above cultures. No IPTG was added to batch cultures and PhoB levels were used for phenotype classification of individual colonies. (C) Population of individual colonies with different PhoB expression phenotypes. Colonies isolated from adapting cultures were classified into three groups by PhoB expression levels (Figure S3). High level corresponds to the autoregulated WT PhoB concentration seen under Pi-deplete conditions and low level is comparable to the basal expression of the original LAC strain. Colonies with intermediate levels substantially different from the above two levels were classified as the medium PhoB expression phenotype. (D) Mapping of adaptive mutations. Colonies with elevated PhoB levels were selected for sequencing of the *lac* promoter and *lacI*. Mutations in *lacO*1 and *lacI* as well as numbers of colonies carrying the particular mutation are indicated. Dots represent identical nucleotides and dashes represent absence of nucleotides. For genotype α, PCR with multiple *lacI*-specific primers failed to yield any products, suggesting the loss of the entire *lacI*, possibly due to deletion. (E) IPTG-induced PhoB levels of selected colonies with different genotypes.

In LAC_0_ cultures, PhoB concentration is extremely limited at the initial stage and cells faced a great challenge of Pi-depletion that drove the evolution. An intermediate concentration (40 µM) of IPTG in continuous cultures induced the PhoB concentration to a moderate and sub-optimal level in LAC_40_ cultures, yet a Pi-deplete environment still prompted evolution of LAC cells, although at a slower pace (Figure 6). Increase of PhoB levels was observed after 48 h of continuous growth in LAC_40_ cultures while only 24 h were required for the LAC_0_ culture to show the first sign of adaptation (Figure 6A). Correspondingly, for individual colonies isolated from 24 h growth samples, all LAC_40_ colonies displayed a low PhoB level in the absence of IPTG similar to that of the original LAC strain while one adaptive mutant with high PhoB expression started to emerge in LAC_0_ cultures (Figure 6C and S3). At 48 h, the majority of LAC_0_ colonies had already adapted with high levels of PhoB comparable to the optimal WT level. At the same time, adaptation was observed in less than half of the LAC_40_ colonies. Further growth until 86 h increased the population of adapted cells in LAC_40_ cultures. Interestingly, most of the adapted LAC_40_ colonies shared a similar phenotype with intermediate uninduced PhoB levels, different from the high optimal PhoB level seen in adapted LAC_0_ cells. Sequencing results revealed that all colonies with high PhoB levels lost functional LacI through deletion, frame-shifting insertion or early termination of *lacI*. In contrast, most of the LAC_40_ colonies with intermediate PhoB levels had an unaltered *lacI* but carried diverse mutations in the LacI repressing site *lacO*1 (Figure 6D). Such mutations relaxed LacI repression and 40 µM IPTG was sufficient to induce the PhoB level to the optimal WT level in these mutants (Figure 6E). Despite different initial conditions and diverse genotypes in adapted cells, evolution in LAC_0_ and LAC_40_ cultures both led to the optimal PhoB concentration that confers maximal fitness to cells in Pi-deplete environments.

### Adaptation under Pi-Replete Conditions

**Figure 7:**
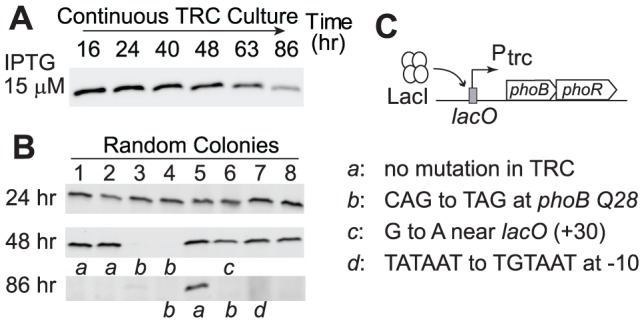
Cells overexpressing PhoB adapt to Pi-replete environments by abolishing PhoB expression. (A) PhoB levels of the adapting culture under the Pi-replete condition. RU1618 (TRC) was induced with 15 µM IPTG to achieve overexpression of PhoB and allowed for adaptation in continuous cultures. (B) PhoB expression in batch cultures from random colonies isolated at different times. Lower case letters indicate genotypes identified in selected colonies that were subjected to sequencing analyses. (C) List of mutations for adapted cells with altered PhoB expression.

As discussed above, a high concentration of PhoB does not provide benefits under Pi-replete conditions and the cost of protein production reduced cell fitness. Therefore, a laboratory evolution experiment similar to LAC_0_ and LAC_40_ continuous cultures was performed to examine whether a strain with a high PhoB level would adapt to Pi-replete environments by reducing PhoB expression, thus increasing its fitness. An IPTG concentration of 15 µM induced a high level of PhoB in the TRC strain and continuous growth in the Pi-replete culture resulted in a gradual decrease of PhoB levels (Figure 7A). Analyses of individual colonies revealed that adapted cells with reduced or abolished PhoB expression already emerged after 48 h of growth (Figure 7B). After 86 h, approximately 30 generations, the majority of colonies isolated from the culture no longer expressed significant amounts of PhoB. Again, different mutational strategies were discovered among these adapted cells that yielded similar phenotypes with reduced PhoB expression (Figure 7C).

## Discussion

Although expression of a significant number of genes is suggested to be non-optimal or even maladaptive under laboratory conditions [26], responsive gene regulation is generally considered to be an evolutionary strategy that allows cells to adjust the levels of beneficial proteins as needed under specific environmental conditions. Costs of gratuitous gene expression and benefits of induced protein production have been characterized for many adaptive genes, particularly those involved in antibiotic resistance or utilization of specific metabolites [27]–[30]. It has been shown that cells can rapidly evolve to different optimal protein levels with balanced fitness costs and benefits under different environments [27], [31]. For the PhoB/PhoR TCS regulators, cost of PhoB/PhoR expression appears to be minimized under Pi-replete conditions via low expression. Under Pi-deplete conditions, benefits derived from the output responses of regulated gene products offset the expression cost and a different optimal PhoB/PhoR concentration has been selected to reach a cost-benefit balance. Autoregulation of PhoB/PhoR expression serves as a mechanism for cells to achieve respective optimal concentration and fitness under these conditions.

### Cost of PhoB/PhoR Expression

Production of unneeded proteins often carries a fitness cost [32]. One of the most extensively studied examples is the *E. coli lac* operon that encodes genes for utilization of lactose. In the absence of lactose, gratuitous induction of the *lac* operon reduces the growth rate and this reduction reflects the deleterious activity of the LacY permease as well as the cost of producing unneeded proteins with cellular resources [27], [28], [30]. Similarly, the fitness cost of high TCS expression can arise from protein production cost and potential detrimental protein activities of RR-regulated genes. It has been shown that constitutive activation of PhoB-regulated genes, particularly the *pst* operon encoding a Pi-transport system, hampers growth under Pi-replete conditions [24], [25]. However, high levels of PhoB/PhoR do not cause significant phosphorylation of PhoB in the absence of stimuli and only a minor basal activation of PhoB-regulated genes were observed at high PhoB levels [19]. Mutation of the conserved D53 residue almost completely abolished the basal activation [19] yet high expression of the mutant gave similar fitness reduction as high expression of intact PhoB/PhoR proteins. Thus detrimental activities of PhoB-regulated genes do not appear to be a major contributor to fitness reduction under the tested Pi-replete conditions although it cannot be excluded that some fitness cost may still originate from residual phosphorylation-independent basal activation of PhoB-regulated genes.

Under Pi-replete conditions, WT *E. coli* cells produce approximately 0.13 pmol of PhoB per 0.3 OD*ml of cells (Table S1), corresponding to a concentration of ∼0.5 µM or 200–300 molecules per cell. Apparently, maintaining such a low level of this 26-kDa protein and an even lower level of PhoR does not have much fitness cost. Increasing expression 50–100 fold in the constitutive TRC strain results in a PhoB concentration of only 25–50 µM. It has been reported that full induction of the *lac* operon yields ∼50 µM LacZ molecules (116 kDa) [33] and causes a 4.5% reduction in batch culture growth rates [27]. Therefore, it is not surprising that a fitness cost of PhoB overexpression was not revealed above the observed 5% data variance of growth rates in batch cultures. In contrast, a gradual fitness reduction caused by PhoB production was apparent in continuous cultures. Because production of useless proteins is an inefficient use of limited resources in nitrogen-limited chemostat cultures, fitness differences likely were magnified. Cost of protein production is clearly dependent on growth conditions and cells tend to reduce the cost with various mechanisms [32], [34], including complete abolishment of expression as observed in our adaptation experiments.

### Cost-Benefit Balance and Evolutionary Optimization of PhoB/PhoR Levels

Pi-depletion leads to an ∼20 fold increase of PhoB level in WT cells. The cost of producing ∼6000 PhoB molecules per cell is compensated by the beneficial function of PhoB/PhoR proteins to provide a close-to-peak fitness. Fitness benefits arise from PhoB∼P-dependent expression of regulated genes, such as the *pst* operon encoding the Pi transporter system and the outermembrane porin gene *phoE*. Although the exact fitness contribution of individual PhoB-regulated genes is difficult to track, the overall fitness landscape correlates well with the output PhoB phosphorylation profile. Peak fitness occurs at a PhoB level close to where PhoB∼P starts to saturate, a point determined by the specific balance of PhoR kinase and phosphatase activities [19]. The constitutive strain in laboratory evolution experiments and the autoregulated WT strain all evolved to express PhoB close to this optimal level for maximal fitness. Above this level, high PhoB levels presumably increase cost without providing further benefits because of the saturation of phosphorylation. RR phosphorylation saturation is not unique to the PhoB/PhoR system but rather a result of the intrinsic HK-RR phosphorylation cycle determined by TCS protein activities [7], [19], [35], [36]. A similar output saturation profile has also been revealed for the autoregulated *E. coli* PhoQ/PhoP system and the induced WT PhoP level is again close to the beginning of saturation [7]. It remains to be investigated whether the expression levels of PhoQ/PhoP and other TCSs are similarly optimized to the phosphorylation output profile.

The laboratory evolution experiments indicated that bacteria with non-ideal TCS expression could increase their fitness through mutations that produced optimal levels of TCS proteins for the specific environment. The majority of mutations were at the promoter of *phoBR* or the regulatory gene of the promoter, suggesting the expression of TCS as a convenient and efficient evolutionary target for environmental adaptation. As the *lac* promoter of *phoBR* in the LAC strain is under negative regulation by LacI, any loss-of-function mutants of *lacI* can result in higher expression of *phoBR* and this may contribute to the rapid evolution pace observed in continuous cultures.

For bacteria with different initial sub-optimal fitness, such as the LAC_0_ and LAC_40_ cultures, adaptation occurred at different rates yet produced similar optimal PhoB expression levels with distinct genotypes. In the absence of IPTG, null *lacI* results in an unrepressed optimal PhoB level that gives adapted LAC cells higher fitness than *lacO* mutants whose PhoB levels are below the optimal level, thus *lacI* null mutants predominate in LAC_0_ cultures. In continuous cultures with 40 µM IPTG, both *lacI* deletion and *lacO* mutations were able to give optimal expression of TCS proteins. All isolated *lacO* mutants carry G:C-to-T:A substitutions and constitute a majority of evolved cells in LAC_40_ cultures. The dominance of *lacO* mutants may reflect a higher mutation frequency for single nucleotide substitution than for frame-shifting insertion or gene deletion observed in *lacI* null mutants. Indeed, nutrient-limited continuous cultures have been known to cause mutations in a mismatch repair gene *mutY*, which greatly increases the frequency of G:C-to-T:A transversions [37]. Despite diverse types of mutations observed in the evolved population, adapted bacteria cells all converged to similar phenotypes in TCS expression to match the demand of the environment.

### Role of Autoregulation in Expressing Optimal PhoB/PhoR Levels

Responsive gene regulation is generally a favored mechanism for adaptation to variable environments with conflicting demands of protein expression. In natural habitats where *E. coli* cells face Pi-rich conditions, such as intestinal lumen, and Pi-limited conditions, such as aquatic environments, Pi-responsive autoregulation of the PhoB/PhoR regulators appears to be an evolutionary consequence that achieves optimal PhoB/PhoR levels under both environments. Under constant environments in continuous cultures, the autoregulated WT strain does not confer apparent fitness advantages over the constitutive strains as long as *phoB* expression matches the WT concentration. However, fixed constitutive expression can only achieve optimal fitness in one particular environment whereas autoregulation gives cells maximal fitness in variable habitats.

Positive autoregulation allows elevated output responses and higher fitness benefits under stimulated conditions. It is this output-amplifying feature of positive autoregulation that enables cells to reduce protein production cost in the absence of stimuli without sacrificing the capacity of output responses. Other features commonly associated with positive autoregulation, such as bistability and response delay, were not observed for the WT PhoB/PhoR system. For strains with non-native genetic background, all-or-none responses have been documented when PhoR is absent and PhoB is cross-phosphorylated by overexpressed non-cognate HKs [38]. However, such cross-phosphorylation is suppressed by the phosphatase activity of the cognate HK and may not be physiologically relevant [19], [39]. Phosphatase-defective or monofunctional HKs are known to promote bistability [7], [40], thus bimodal responses in these non-native cross-talk systems may arise from the lack of negative phosphatase activity in non-cognate HKs [41]. Positive autoregulation has been shown advantageous for the *Salmonella* PhoQ/PhoP system to promote virulence in mice [42]. The fitness gain over the constitutive strain was suggested to be associated with a transient surge in RR phosphorylation observed for the autoregulated strain even though the activation surge was later attributed to intrinsic negative feedback of biochemical activities in bifunctional HKs [8], [43]. It is possible that part of the fitness advantages for the autoregulated PhoQ/PhoP system may come from lowered costs in unstimulated environments, similarly to fitness profiles observed for the PhoB/PhoR system. Balancing costs and benefits by positive autoregulation may be a recurring scheme in TCSs to select proper TCS protein quantities and biochemical activities.

## Materials and Methods

### Strains and Growth Conditions

The strains and plasmids used in this study are listed in Table S2. λ red recombination [44] was used to make chromosomal gene disruption or alteration in strain BW25113 or derivatives of BW25113 similarly to the constitutive strains, RU1616 (LAC), RU1617 (KON) and RU1618 (TRC), in which the WT autoregulated phoB promoter was replaced with constitutive (KON) or IPTG-inducible (LAC and TRC) promoters [19]. Strains with the chromosomal reporter *phoA*-*yfp* or YFP marker were created using the reported recombination strategies [45]. Briefly, the plasmid pRG261 containing *P_phoA_-yfp* reporter or pRG278 containing P*_tet_*-*yfp* was integrated into the chromosome of indicated strains at the HK022 or lamda phage attachment sites to generate RU1465 (WT, *phoA-yfp*), RU1653 (LAC, *phoA-yfp*) and RU1622 (WT-*yfp*), respectively. Details of strain and plasmid construction were described in Text S1.

Bacteria batch cultures were grown in MOPs minimal media [46] with 0.4% glucose, containing either 2 mM (Pi-replete) or 50 µM (Pi-deplete) KH_2_PO_4_. Continuous cultures were grown at 37°C in a home-built chemostat modified from the design described in [47]. Fresh feed medium was supplied to a 50-ml glass vessel through silicone tubing with a peristaltic pump at a flow rate of 6 ml/h while bacteria culture flowed out through an outflow tube at the same rate. The total culture volume is 24 ml, set by the depth of the outflow tube in the chemostat vessel. Thus the dilution rate was 0.25 h^−1^, corresponding to a generation time of ∼2.8 h. The feed medium was identical to the MOPs medium used in batch cultures except that the concentration of NH_4_Cl was limited at 250 µM instead of 5 mM in batch cultures. Optical densities (600 nm) of the nitrogen-limited chemostat were ∼0.09, corresponding to ∼2.2×10^9^ cells in the chemostat culture. Pi concentrations of 300 µM and 12 µM were included in feed media for Pi-replete and -deplete conditions, respectively.

### YFP Reporter, AP Activity and Protein Level Measurements

As described previously [19], to assay bacterial responses to phosphate concentrations, cells from fresh Pi-replete MOPs cultures were inoculated in MOPs medium containing 2 mM (Pi-replete), 50 µM (Pi-deplete) or indicated concentrations of KH_2_PO_4_ with a starting OD_600_ at 0.04 followed by 3 h growth. For YFP reporter assays, fluorescence of individual cells was measured with a Beckman Coulter FC500 flow cytometer. Mean fluorescence of the whole population was calculated from the fitted lognormal distribution. For AP activities and protein level determination, bacteria pellets equivalent to 0.3 OD_600_*ml from batch or continuous cultures were collected and assayed as described before [19]. AP activities were determined by monitoring the rate of absorbance change at 420 nm using a microplate reader (Varioskan, ThermoFisher) following addition of 7 mM *p*-nitrophenylphosphate. Values of absorbance changing rates were multiplied by 100000 to represent absolute AP activities while they are compared to AP activity of the WT strain for relative AP activities. PhoB levels from sample lysates were determined by western blot. Blots probed with anti-PhoB primary antisera and Cy5-conjugated secondary antibodies were visualized by fluorescence imaging with a FluorChem Q (Alpha Innotech). Selected blots were simultaneously probed with the anti-Sigma70 and Cy3-conjugated secondary antibodies to confirm equal loading of samples.

### Cell Fitness Assays

Cell fitness was evaluated with growth rates in batch cultures and competition assays in continuous cultures. Indicated strains were induced with indicated IPTG concentrations in MOPs media for 2–3 h to achieve different PhoB/PhoR levels. Cells from these fresh MOPs cultures were inoculated in 96-well plates and grown at 37°C with a starting OD_600_ of ∼0.03 under respective IPTG conditions. The exponential growth rates before Pi-depletion were determined by fitting the data with a single exponential function.

Competition assays were performed in continuous cultures with the indicated non-fluorescent strains and WT-*yfp*. Cells from fresh MOPs cultures were mixed at a 1∶1 ratio and inoculated into the chemostat with a starting OD_600_ of ∼0.04. Indicated IPTG concentrations were included in the feed medium to achieve different PhoB levels. After 24 h of growth, cells collected from the outlet stream were analyzed by microscope imaging or flow cytometry to determine the population of individual strains. Bacteria cells were immobilized on 1% agarose pads made with MOPs medium as described [48]. Microscope images were obtained using an Olympus IX70 microscope (Olympus) with a 100× NA PlanApo 1.3 objective, 100 W mercury lamp and the HiQ fluorescein filter set (Chroma). Phase-contrast images were taken to determine the total cell numbers and fluorescent images were used to count the population of fluorescent WT-*yfp* cells. Cell numbers were counted using ImageJ software (NIH) and bacteria population was determined from at least 1000 cells from a total of 6–10 images. In selected samples, cell populations measured by microscope imaging were confirmed by flow cytometry.

### Adaptation Experiments in Continuous Cultures

Indicated bacteria strains were grown in continuous cultures for ∼4 days. Pi concentrations of 300 µM (Pi-replete) and 12 µM (Pi-deplete) were included in feed media together with indicated concentrations of IPTG to yield non-optimal PhoB expression levels under different Pi conditions. Cells were collected from the outlet stream at indicated time intervals. Collected cultures were pelleted and 0.3 OD_600_*ml of cells were stored at −80°C for later examination of AP activities and PhoB levels. Diluted cultures from the chemostat were streaked on LB plates for colony isolation. Single colonies were randomly chosen to grow in MOPS batch cultures and characterized for their phenotypes in PhoB expression and AP activities. To investigate the genotype of individual clones, colony PCR was performed to amplify the chromosomal DNA regions corresponding to the *lacI* gene, *phoB* and its promoter. Sequences of these regions were determined and compared to the WT sequence. For some colonies, primers specific to *lacI* yielded no PCR products and further use of additional primers corresponding to different coding, upstream and downstream regions of *lacI* still did not give PCR products, suggesting the loss of *lacI*.

## Supporting Information

Figure S1 Pi concentration dependent *phoA* reporter activities. (A) Identical AP activity profiles of WT (BW25113) and *phoA-yfp* carrying WT strain (RU1465). Error bars are SDs of three independent experiments and unseen error bars are smaller than symbols. (B) Population distribution of single-cell output responses of the constitutive LAC strain (RU1653) at indicated initial Pi concentrations. IPTG concentrations of 150 µM (upper) and 0 µM (lower) were used to give high and low PhoB expression levels comparable to the induced autoregulated and uninduced basal WT levels. Solid lines represent lognormal fit of population distribution.
(EPS)

Figure S2 AP activities of WT cells in continuous cultures. Different Pi concentrations in inlet MOPs media were used to evaluate Pi-replete and -deplete conditions in continuous cultures. Cells consumed phosphates for growth and Pi concentrations in outlet cultures of first three samples (12, 14, 18 µM inlet Pi concentrations) were below the detection limit, thus only the Pi concentrations in inlet fresh media were shown. Error bars are SDs of at least three independent experiments.
(EPS)

Figure S3 PhoB expression levels of individual colonies probed by immunoblot. Colonies isolated at different time from LAC_0_ (A, B, C) and LAC_40_ (D, E, F) cultures were grown in MOPs media (Pi-replete, 2 mM Pi) and assayed for PhoB levels. L, M and H labels below each blot indicate arbitrary assessment of low, intermediate and high phoB expression levels. Immunoblot assays were repeated at least twice for each colonies and only one representative set is shown.
(EPS)

Table S1 Competition results between WT-*yfp* and non-fluorescent strains. Quantification values are shown as the mean ± SD. “*n*” represents the number of independent experiments. PhoB protein levels were determined previously from 0.3 OD·ml of cells
(DOC)

Table S2 Strains and plasmids used in this study.
(DOC)

Text S1 Cloning of strains and plasmids.
(DOC)
