# Supplementary material for: Evolutionary Tuning of Protein Expression Levels of a Positively Autoregulated Two-Component System
Source: PLoS Genet. 2013 Oct 24;9(10):e1003927. doi: 10.1371/journal.pgen.1003927 (PMC3812086; doi:10.1371/journal.pgen.1003927)
Supplement: Table S1 — Competition results between WT-yfp and non-fluorescent strains. Quantification values are shown as the mean ± SD. “n” represents the number of independent experiments. PhoB protein levels were determined previously from 0.3 OD·ml of cells (DOC) [file pgen.1003927.s004.doc]

Table S1. Competition results between WT-*yfp* and non-fluorescent strains.

| Competitors  v.s.WT-*yfp* | PhoB Amount (pmol)* | Population after 24 hr % | | | |
| --- | --- | --- | --- | --- | --- |
|  |  | Pi-deplete | *n* | Pi-replete | *n* |
| WT (BW25113) |  |  |  |  |  |
| Pi-replete | 0.13 | NA |  | 50 ± 2 | 3 |
| Pi-deplete | 2.79 ± 0.46 | 50 ± 2 | 2 | NA |  |
| Δ *phoB* | 0 | 1 ± 1 | 2 | 52 | 1 |
| LAC w/IPTG |  |  |  |  |  |
| 0 μM | 0.13 | 7 ± 5 | 5 | 48 ± 3 | 4 |
| 25 μM | 0.73 ± 0.20 | 8 ± 1 | 3 | 49 ± 1 | 3 |
| 50 μM | 1.44 ± 0.24 | 22 ± 9 | 3 | 42 ± 6 | 3 |
| 150 μM | 2.45 ± 0.37 | 50 ± 10 | 3 | 45 ± 10 | 3 |
| TRC w/IPTG |  |  |  |  |  |
| 0 μM | 5.56 ± 0.64 | 56 ± 1 | 2 | 34 ± 1 | 2 |
| 5 μM | 8.85 ± 0.52 | 40 ± 2 | 2 | 26 ± 1 | 2 |
| 15 μM | 14.5 ± 1.4 | ND |  | 22 ± 1 | 2 |
| KON | 10.1 ± 1.0 | 15 ± 1 | 3 | 23 ± 6 | 3 |

Quantification values are shown as the mean ± SD. “*n*” represents the number of independent experiments.

* PhoB protein levels were determined previously from 0.3 OD·ml of cells [1].

NA, Not Available ND, Not Determined.

**Reference**

1. Gao R, Stock AM (2013) Probing kinase and phosphatase activities of two-component systems *in vivo* with concentration-dependent phosphorylation profiling. Proc Natl Acad Sci USA 110: 672-677.
