# Supplementary material for: Evolutionary Tuning of Protein Expression Levels of a Positively Autoregulated Two-Component System
Source: PLoS Genet. 2013 Oct 24;9(10):e1003927. doi: 10.1371/journal.pgen.1003927 (PMC3812086; doi:10.1371/journal.pgen.1003927)
Supplement: Table S2 — Strains and plasmids used in this study. (DOC) [file pgen.1003927.s005.doc]

Table S2. Strains and plasmids used in this study.

| Strains / plasmids | Relevant characteristics | Reference / source |
| --- | --- | --- |
| Strains *E. coli* |  |  |
| DH5α | General cloning strain | Invitrogen |
| BW25113 | WT*, lacI*  *rrnB*_T14_ Δ*lacZ*_WJ16_ *hsdR514*  Δ*araBA-D*_AH33_  Δ*rhaBAD*_LD78_ | (1) |
| BW25142 | *pir-116 rrnB3* Δ*lacZ4787* *hsdR514*  DE(*araBAD*)567 DE(*rhaBAD*)568 Δ*phoBR580* | (2) |
| RU1465 | *attHK::*pRG261(*PphoA-yfp*) in BW25113 | This study |
| RU1616 | LAC, Φ(Δ*phoBp* P_lac_-*phoBR*) in BW25113 | (3) |
| RU1617 | KON, Φ(Δ*phoBp* *phoBR*) replacement of phoB box with -35 sequence in BW25113 | (3) |
| RU1618 | TRC, Φ(Δ*phoBp* P_trc_-*phoBR*) in BW25113 | (3) |
| RU1619 | KON(D53A), Φ(Δ*phoBp* *phoB^D53A^R*) | This study |
| RU1622 | WT-*yfp*, *attλ::*pRG278(*Ptet-yfp*) in BW25113 | This study |
| RU1631 | Δ*phoB* in BW25113 | (3) |
| RU1653 | LAC (*phoA-yfp*) *attHK::*pRG261(*PphoA-yfp*) Φ(Δ*phoBp* P_lac_-*phoBR*) | This study |
| RU1722 | Δ*lacI* in LAC (RU1616) | This study |
| RU1723 | LAC* (IV), Φ(P_lac_(*lacO**)-*phoBR*) | This study |
|  |  |  |
| Plasmids |  |  |
| pET21b | T7 polymerase-based expression vector, Ap ^r^ | Novagen |
| pAH63 | CRIM plasmid for integration at *attλ* site, Km^r^ | (2) |
| pAH144 | CRIM plasmid for integration at *attHK* site, Sp^r^ | (2) |
| pCL1920 | low copy number plasmid, pSC101 origin, Sp^r^ | (4) |
| pJZG146 | *rrnB*-MCS-*mYFP* in pCL1920, Sp^r^ | This study |
| pRG2 | *lacI^q^* *P_lac_-phoB*, Ap ^r^ | (5) |
| pRG161 | *PphoA*-*mYFP* in pJZG146, Sp^r^ | This study |
| pRG177 | *lacI^q^* *P_lac_-yfp*, pRG2 derivative, Ap ^r^ | This study |
| pRG252 | *Ptet* promoter, Ap ^r^ | This study |
| pRG261 | *PphoA*-*mYFP* in pAH144 for CRIM integration, Sp^r^ | This study |
| pRG276 | *Ptet-yfp*, pRG252 derivative, Ap ^r^ | This study |
| pRG278 | *Ptet-yfp* in pAH63 for CRIM integration, Km^r^ | This study |
|  |  |  |

**Reference**

1. Datsenko KA, Wanner BL (2000) One-step inactivation of chromosomal genes in *Escherichia coli* K-12 using PCR products. *Proc Natl Acad Sci U S A* 97:6640-6645.

2. Haldimann A, Wanner BL (2001) Conditional-replication, integration, excision, and retrieval plasmid-host systems for gene structure-function studies of bacteria. *J Bacteriol* 183:6384-6393.

3. Gao R, Stock AM (2013) Probing kinase and phosphatase activities of two-component systems *in vivo* with concentration-dependent phosphorylation profiling. *Proc Natl Acad Sci USA* 110:672-677.

4. Lerner CG, Inouye M (1990) Low copy number plasmids for regulated low-level expression of cloned genes in *Escherichia coli* with blue/white insert screening capability. *Nucleic Acids Res* 18:4631.

5. Gao R, Tao Y, Stock AM (2008) System-level mapping of *Escherichia coli* response regulator dimerization with FRET hybrids. *Mol Microbiol* 69:1358-1372.
