# Supplementary material for: Evolutionary Tuning of Protein Expression Levels of a Positively Autoregulated Two-Component System
Source: PLoS Genet. 2013 Oct 24;9(10):e1003927. doi: 10.1371/journal.pgen.1003927 (PMC3812086; doi:10.1371/journal.pgen.1003927)
Supplement: Text S1 — Cloning of strains and plasmids. (DOC) [file pgen.1003927.s006.doc]

**Text S1**

**Cloning of strains and plasmids.**  The strains and plasmids used in this study are listed in Table S2. λ red recombination [1] was used to make chromosomal gene disruption or alteration in strain BW25113 or derivatives of BW25113 similarly to the constitutive strains, RU1616 (LAC), RU1617 (KON) and RU1618 (TRC), in which the WT autoregulated phoB promoter was replaced with constitutive or IPTG-inducible promoters [2]. To construct RU1619, a PCR fragment containing the kanamycin resistance Km^r^ cassette and a *phoB* gene carrying the D53A mutation as well as a -35 consensus sequence (TTGACA) at the promoter was created with recombinant PCR as described before using the following primers, RG147 (5’- CTGCGCCACGGAAATCAATAACCTGAAGATATGTGCGAC GTGTAGGCTGGAG CTGCTTCG -3’), PhoBD53A-r (5’-AGGTAACATCCAGGCGAGGAGAATTAAATC-3’), PhoBD53A-f (5’-GATTTAATTCTCCTCGCCTGGATGTTACCT-3’) and PhoBR-r (5’-TTCCACGACAGCCGTTCCAGC-3’). The PCR product was electroporated into RU1631 for λ red recombination. The Km^r^ cassette was subsequently removed by FRT recombination using pCP20 to give RU1619.

To identify *lac* mutations discovered in laboratory evolution experiments, primers RG166 (5’- GCGAGAGCTCGCATGGGCGCGATTATACC-3’) and #175 (5’-GCCGAAGCTTCGAAATACGGCGCATTACCGC-3’) were used to amplify *phoB* gene and its promoter while RG186 (5’-GGCATGCATTTACGTTGACACCATCGA-3’) and RG195 (5’-CTGCCCGCTTTCCAGTCGGGAAACC-3’) were used to amplify *lacI* followed by sequencing analyses. For some mutants that RG186 and RG195 did not yield any PCR products, multiple combination of primers corresponding to different upstream, downstream or coding regions of *lacI*, including RG194 (5’-ATGGCGGAGCTGAATTACATTCCC-3’), RG243 (5’-CATGCCCGAATGTGCACCAGGTGCAC-3’), RG244 (5’-GCGCAGTGAACAACGGGTGA TTGGCTG-3’) and RG187 (5’-GGTGCCTAATGAGTGAGCTAACTCAC-3’), still yield no PCR products, suggesting a large scale deletion of *lacI*. To recreate *lacI* deletion, primers RG227 (5’-GATGGCGGAGCTGAATTACATTCCCAACCGCGTGGCACGTGTAGGCTGGA GCTGCTTCG-3’) and RG228 (5’-GCTCACTGCCCGCTTTCCAGTCGGGAAACCTGTCGT GCCAATTCCGGGGATCCGTCGAC-3’) were used to amplify the Km^r^ cassette to knock out *lacI* in RU1616 by λ red recombination. Similarly, a PCR fragment containing the Km^r^ cassette and the underlined *lacO* mutation (AATTATGAGCGGATAACAATTT) at the *lac* promoter of *phoB* was generated by recombinant PCR using RG147, RG149 (5’-TCACTGCCCGCTTT CCAGATTCCGGGGATCCGTCGAC-3’), RG150 (5’- GTCGACGGATCCCCGGAATCTGG AAAGCGGGCAGTGA-3’) and #175, followed by λ red recombination in RU1631. The resulting strains RU1722 and RU1723 were confirmed by sequencing analyses of targeted regions. To supply extra copies of *lacI* from plasmid, a pRG2 derivative plasmid containing *lacI^q^*, pRG177, was created by replacing *phoB* between *Xba*I and *Hind*III sites in pRG2 with *yfp* to avoid complication by plasmid borne *phoB*.

Strains with the chromosomal reporter *phoA*-*yfp* or YFP marker were created using the reported recombination strategies [3]. In detail, the promoter of *phoA* was amplified with RG84 (5’-GCACTGCAGGCAATGCTTCGCAATATGGC-3’) and RG63 (5’-GCCTCTAGATCAGT CCGGGCTTTTGTCACA-3’) from *E. coli* chromosome, followed by digestion with *Pst*I and *Xba*I and cloned into a pCL1920 derivative, pJZG146 that contains a promoter-less *yfp*. The fragment containing *phoA-yfp* was then released from the resulting pRG161 plasmid by *Pst*I/*Kpn*I digestion and inserted into pAH144 to give pRG261. The entire plasmid pRG261 was integrated into the chromosome of BW25113 or RU1616 by site-specific recombination at the HK022 phage attachment site with described protocols [3] to generate RU1465 and RU1653, respectively. For a constitutively expressed YFP marker, a *tet* promoter was constructed by annealing oligos RG138 (5’-CTTGTTGACACTCTATCATTGATAGAGTTATTTTACCACTCCCT-3’) and RG139 (5’-CTAGAGGGAGTGGTAAAATAACTCTATCAATGATAGAGTGTCAACAAGCATG-3’) and ligating into the *Sph*I/*Xba*I digested pET21b to give pRG252. A *yfp* gene was released from pRG177 by *Xba*I/*Hind*III digestion and ligated into pRG252 to create pRG276. A fragment containing P*tet*-*yfp* was subsequently cut with *Sph*I and *Sca*I, followed by ligation with *Sph*I/*Sma*I digested pAH63 plasmid to yield pRG278. pRG278 was then integrated into the chromosome of BW25113 at the lamda phage attachment site to generate RU1622. All integration strains were confirmed to have single insertion by the reported PCR methods [3].

**References**

1. Datsenko KA, Wanner BL (2000) One-step inactivation of chromosomal genes in *Escherichia coli* K-12 using PCR products. Proc Natl Acad Sci U S A 97: 6640-6645.

2. Gao R, Stock AM (2013) Probing kinase and phosphatase activities of two-component systems *in vivo* with concentration-dependent phosphorylation profiling. Proc Natl Acad Sci USA 110: 672-677.

3. Haldimann A, Wanner BL (2001) Conditional-replication, integration, excision, and retrieval plasmid-host systems for gene structure-function studies of bacteria. J Bacteriol 183: 6384-6393.
